# Emergence from torpor rapidly elevates suppressed blood immune parameters in a bat species hibernating in a moderate climate

**DOI:** 10.1242/jeb.251962

**Published:** 2026-05-11

**Authors:** Anna Langguth, Nicholas C. Wu, Tomás Villada-Cadavid, Laura A. Brannelly, Jasmin Hufschmid, Gábor Á. Czirják, Christopher Turbill

**Affiliations:** ^1^ One Health Research Group, Melbourne Veterinary School, University of Melbourne, Werribee, VIC 3010, Australia; ^2^ Hawkesbury Institute for the Environment, University of Western Sydney, Sydney, NSW 2753, Australia; ^3^ Centre for Terrestrial Ecosystem Science and Sustainability, Harry Butler Institute, Murdoch University, WA 6150, Australia; ^4^ School of Environmental and Conservation Science, Murdoch University, WA 6150, Australia; ^5^ Department of Wildlife Diseases, Leibniz Institute for Zoo and Wildlife Research, Berlin 10315, Germany; ^6^ School of Science, Western Sydney University, Sydney, NSW 2751, Australia

**Keywords:** Hibernation, Arousal, Immune function, Australian bats, Hibernation physiology, White-nose syndrome

## Abstract

Torpor is a state of reduced metabolism that allows animals to conserve energy during periods of limited resources. Critical physiological processes, including the immune function, are downregulated during torpor – a phenomenon that remains relatively understudied in bats, especially in the southern hemisphere. This study examined the effects of torpor on the immune system of the eastern bent-winged bat (*Miniopterus orianae oceanensis*), an Australian cave-roosting species. We captured 52 bats in austral autumn and winter and housed them in controlled conditions to induce torpor for 2 or 8 h. Blood samples were collected pre-torpor, at the end of the torpor bout and 30 min post-arousal. White blood cell count was measured to assess cellular immunity, and plasma antibacterial capacity was used to evaluate humoral innate antibacterial immunity across time points. We found that total white blood cell count decreased by 23% during torpor, but increased by 75.9% upon arousal, surpassing baseline values by 33.9%. Neutrophils and monocytes were the first cells to be restored, the former making up about 57% of circulating white blood cells after arousal. Antibacterial capacity did not differ between time points. Our results demonstrate rapid restoration of white blood cells after arousal, likely through the release of sequestered immune cells, while short torpor bouts do not impair innate humoral immunity in eastern bent-winged bats. While the rapid restoration of neutrophils could be protective against pathogens accumulated during torpor, it also provides a higher potential for immunopathology and tissue damage upon arousal.

## Teaser

**Summary:** Torpor bouts and season alter immune cell dynamics and innate humoral antibacterial activity in a bat species hibernating in mild winter climates, offering new insights into energy–immunity trade-offs with relevance for bat conservation.

## INTRODUCTION

Torpor is a state of markedly lowered metabolic function and body temperature employed by various bird and mammal species to conserve energy during periods of harsh environmental conditions or limited food availability (Geiser, 2004; Bouma et al., 2010a; Ruf and Geiser, 2015). In mammals, torpor plays a key role during winter hibernation, when minimising energy expenditure is essential for survival (Carey et al., 2003; Bouma et al., 2010b; Sahdo et al., 2013). Hibernation during the colder months of the year comprises a series of extended torpor bouts interspersed with rewarming to periods of normothermia, i.e. regular body temperature (Willis, 1982; Park et al., 2000; Prendergast et al., 2002), with the duration of both phases varying greatly among different climates and across species depending on traits such as body size and metabolic rate (French, 1985; Geiser, 2004; Jackson et al., 2022). Torpor is beneficial to survive periods of seasonal or acute food scarcity (Wu et al., 2025) because insectivorous bats have finite energy reserves to rely on relative to their small body size (Geiser, 2006; Stawski et al., 2014) and high metabolic costs of flight to refuel (Thomas and Suthers, 1972). During torpor, the body temperature of bats can fall to a minimum of 2 to 5°C, nearing ambient temperatures, and their metabolism can be as low as 3–4% of basal metabolic rate (Geiser, 2006). The torpor-induced reduction in physiological activity is accompanied by a downregulation of key metabolic functions, which includes the immune system (Bouma et al., 2010a,b). Studies of hibernating mammals have found a drastic reduction in circulating leukocytes, which decrease by over 90% during torpor in hedgehogs (*Erinaceus europaeus*) (Suomalainen and Rosokivi, 1973), hamsters (*Cricetus cricetus*) (Reznik et al., 1975) and thirteen-lined ground squirrels (*Ictidomys tridecemlineatus*) (Bouma et al., 2010b). It is thought that these cells are largely being sequestered (withdrawn from circulation and stored elsewhere) or marginated (migration to the walls of blood vessels) to conserve energy (Bouma et al., 2013a). While a lower body temperature naturally slows cell metabolism, the sequestration of white blood cells (WBCs) also reduces their activity, prevents unnecessary responses and allows them to remain in a low-energy state (Szilagyi and Senturia, 1972; Inkovaara and Suomalainen, 1973; Yasuma et al., 1997). Torpor not only alters cellular components of the immune system but also affects factors within the plasma. These include complement proteins that help target and destroy pathogens (Maniero, 2002), and cytokines, which are signalling molecules that coordinate immune responses (Bouma et al., 2013b). While these factors are not actively removed from circulation during torpor and do not require energy expenditure to persist, their production and biochemical function are nonetheless strongly reduced (Maniero, 2002).

In northern hemisphere species, the overall length of the winter season (Graesli et al., 2015; Kizhina et al., 2018) has been shown to suppress immune function, and the duration of individual torpor bouts has been linked to progressive changes in immune cell composition (Huber et al., 2021). However, the extent to which these dynamics apply to species inhabiting milder climates, or to those that employ shorter or more variable torpor bouts, remains poorly understood. Addressing this knowledge gap offers an opportunity to identify periods of increased vulnerability to emerging diseases in southern hemisphere populations and to inform targeted disease mitigation strategies. By examining blood immune parameters, namely absolute and differential WBC count and antibacterial capacity of plasma before, during and after arousal from torpor under controlled laboratory conditions, this study aimed to understand how the physiological states of torpor and arousal affect immunological potential in an insectivorous southern hemisphere bat species hibernating in winter. This paper describes the effects of short torpor bouts in the eastern bent-winged bat (*Miniopterus orianae oceanensis*), an insectivorous Australian hibernator considered at high risk of future exposure to *Pseudogymnoascus destructans* (Holz et al., 2019; Turbill and Welbergen, 2020), the pathogen causing the potentially fatal ‘white-nose syndrome’.

## MATERIALS AND METHODS

We trapped adult eastern bent-winged bats (*Miniopterus orianae oceanensis* (Cardinal and Christidis 2000); *n*=64; 40 males, 24 females) during austral autumn (May) and winter (July) 2024 at the Jenolan Karst Conservation Reserve, NSW, Australia (approximately 33°49′S, 150°01′E). Animals were captured during flyout, using a harp trap placed at the cave entrance. Once the bats were captured, we determined sex by assessing external genitalia. We also measured the right forearm to the nearest 0.1 mm with digital callipers (B09QL1RB1G, Ziyuan Electronics Co., Ltd, Guangdong, China) and assessed body mass to the nearest 0.1 g with a digital scale while on site (MS500, Pesola Präzisionswaagen AG, Schindellegi, Switzerland). Only animals weighing more than 13 g were included in experiments. After capture, animals were transported approximately 2 h to the Hawkesbury Campus of Western Sydney University, where they were held for up to 3 days before being released at their original capture site. The experiment was run two separate times, with 44 individuals enrolled in autumn and 20 individuals captured for experiments in winter.

Animals were placed in individual respirometry chambers (1.3 l; Tritan Ultra Square, Sistema, Auckland, New Zealand) lined with absorbent paper at the bottom and plastic mesh on the sides. Chambers were housed within an incubator (749 l; IPP750plus, Memmert GmbH+Co. KG, Schwabach, Germany) set to 8°C, and relative humidity was maintained above 95%, both approximating natural cave conditions that our study species would experience (Dwyer, 1964), using a combination of wet towels and pre-humidified airflow. Ambient air was pumped into the incubator with a diaphragm vacuum pump (N920KT.29.18, KNF Neuberger, Inc., Trenton, NJ, USA) at a flowrate of 8–12 l min^−1^ and humidified by bubbling through a 2 l water bottle. Individual metabolic rates were monitored to determine periods of torpor use during each experimental run by pull air-flow respirometry (chamber air flow=1 l min^−1^) using the Promethion Core System (Sable Systems International, North Las Vegas, NV, USA). This system provided real-time measurements of oxygen, carbon dioxide and humidity in each animal chamber. Bats were placed in individual respirometry chambers at the start of each experimental run and continuously monitored to determine the timing of torpor entry. Torpor patterns were recorded remotely via the Promethion system. Animals were classified as normothermic when CO_2_ emission exceeded 0.5 ml min^−1^. Based on the mean body mass of the study animals (14.85 g), this corresponds to an oxygen consumption rate of approximately 2 ml O_2_ g^−1^ h^−1^. This threshold was based on previous studies indicating that torpid insectivorous bats of similar body size typically exhibit O_2_ consumption rates well below this value, often under 0.1–0.2 ml min^−1^ at an air temperature of around 8°C (Hanus, 1959; Willis et al., 2005). Values above 2 ml O_2_ g^−1^ h^−1^ are consistent with normothermic metabolic rates from Hanus (1959) and Willis et al. (2005) and are therefore indicative of wakefulness. Once an animal entered torpor, it was maintained in that state for either 2 or 8 h, depending on its randomly assigned treatment group. Based on a pilot study, the 8 h time frame was the longest bats would reliably remain in torpor within our setup, while the 2 h time frame was chosen as the maximum amount of time animals needed to reach steady-state torpor. Of the 64 individuals enrolled in the experiment, 12 did not enter torpor at any point. The final group sizes therefore included 17 bats in autumn and 12 in winter for the 8 h category, while the 2 h category included 15 bats in autumn and 8 in winter.

We collected 40 µl of blood at three different time points per bat, allowing us to follow individual parameters: pre-torpor (baseline), during torpor (at the end of a pre-determined torpor bout while torpid) and post-torpor (30 min after arousal). All blood samples were collected into heparinised 75 µl microcapillary tubes. A blood smear was prepared from all samples (see below) and capillary tubes were centrifuged at 1000 ***g*** for 5 min, after which plasma and the cell pellet were separated into individual Eppendorf tubes and stored at −80°C. Each animal was weighed before collection of each of the three blood samples.

Upon arrival of the bats at the lab, a baseline blood sample (*n*=52) was collected from the saphenous vein within the uropatagial membrane by placing the bat into dorsal recumbency on top of a calico bag and gently extending the uropatagial membrane. The membrane was disinfected with 80% ethanol, and a thin layer of petroleum jelly was applied to ensure that blood pooled on the wing membrane surface. The vein was punctured using a 26-gauge needle and blood droplets were collected. Immediately after collection of the baseline blood sample, bats were placed in individual respirometry chambers and into the incubator.

After either 2 or 8 h of continuous torpor, bats were removed from the incubator for collection of a second blood sample, which was taken while they were still in torpor. To prevent arousal, animals were immediately placed under short-term anaesthesia, using a Stinger mobile gas inhalation anaesthesia machine (DarvallVet, Gladesville, NSW, Australia) and a zero dead-space circuit and mask, administering 3–5% isoflurane vapourised in oxygen at a flow rate of 500 ml min^−1^. After checking for appropriate anaesthetic depth via toe-pinch, blood was then collected from the jugular vein. This location was chosen as an established alternative to peripheral vein sampling (Fritze et al., 2021), which is not feasible during torpor because of reduced peripheral blood flow. Animals were positioned in dorsal recumbency, and a heparin-coated 27-gauge 1 ml insulin syringe was inserted between the head and the shoulder of the bat, parallel to the body of the animal, aiming towards the sternum. After the needle was inserted, it was slowly pulled back while applying negative pressure to the syringe until blood was seen pooling in the needle conus. After 40 µl of blood was collected, the sample was immediately transferred into a heparinised microcapillary tube. This second sample was collected from a total of 33 torpid individuals. Nineteen individuals were excluded from the ‘torpor’ time point because blood collection was unsuccessful after three attempts and these bats were allowed to arouse without the sample being collected; all animals subsequently recovered to normal physiological states.

Following this process, bats were placed in individual boxes and allowed to arouse from torpor following the removal of isoflurane. A third blood sample was collected from the saphenous vein (*n*=47) 30 min after the onset of arousal. This final sample was not collected from five individuals that had a body mass below the minimum limit. Supplementary feeding with mealworms was provided to all bats both after the last blood sample was collected and before they were released. Bats were released at the original capture site, just after sunset on the day following collection of the final blood sample in each group.

All trials were approved by Western Sydney University's Animal Care and Ethics Committee (A15746) and by a New South Wales scientific licence under the Biodiversity Conservation Act 2016 (SL102862).

### WBC count

Blood smears were made from samples collected at each time point. Smears were air-dried and then stained using a Three-Step Stain Kit (IS3300, Thermo Fisher Scientific, Waltham, MA, USA). Slides were manually examined using light microscopy (Olympus BX41, Olympus Corporation, Tokyo, Japan) under high-powered fields (HPFs) at 40× magnification. Total WBC count was obtained by scanning 10 HPFs at 40× magnification. The total leukocyte count was then calculated by dividing the number of leukocytes observed by the number of view fields scanned and multiplying this ratio by a commonly used correction factor of 2 to estimate the approximate number of leukocytes per litre of blood (Voigt and Swist, 2011; Moore et al., 2013). Differential counts included neutrophils (segmented and band neutrophils), lymphocytes, monocytes, eosinophiles and basophiles and were obtained by identifying and classifying 100 leukocytes per slide.

### Assessment of plasma antimicrobial capacity

To assess humoral innate immunity, i.e. components of the immune system present in blood plasma, bacterial killing assays were performed using *Escherichia coli* (ATCC #8739), following a published protocol (Liebl and Martin, 2009). This assay tests to what degree humoral innate immune components can inhibit the growth of a model pathogen within a given amount of time. To do this, each plasma sample was diluted 1:8 (2 µl sample to 16 µl PBS as determined by preceding serial dilution trials) and added in triplicate to a 384-well plate (M1937-32EA, Merck Life Science Pty Ltd, Bayswater, VIC, Australia). Subsequently, 6 µl of bacterial solution (10^5^ colony-forming units per millilitre) was added to each well. A sample blank with the same dilution was also plated with 6 µl PBS instead of bacterial solution. We further plated 18 µl PBS with 6 µl bacteria as a positive control, and 24 µl PBS as a negative control (blank). After shaking on a plate-shaker for 1 min, plates were incubated for 30 min at 37°C. We then added 125 µl of Tryptic Soy Broth (41298, Merck Life Science Pty Ltd) to all wells, returned plates to the shaker for another minute, and then incubated plates at 37°C for 12 h overnight. The absorbance in each well was then read at 300 nm using a spectrophotometer (Varioskan™ LUX, Thermo Fisher Scientific, Waltham, MA, USA). The index of bacterial killing was calculated as:
(1)

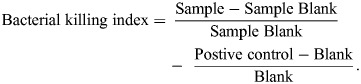


### Statistical analysis

Data analysis was carried out using R studio version 4.4.1 (https://www.r-project.org/). A significance threshold of *P=*0.05 was used as a cutoff for all analyses. We analysed the data using linear mixed effects models (LME; ‘lme4’ package; Bates et al., 2015) to assess the impact on total WBC count, neutrophil-to-lymphocyte ratio (NLR) (both natural log transformed) and plasma bacterial killing index. Generalised linear mixed effects models (GLME; ‘glmmTMB’ package; Brooks et al., 2017) with a gamma distribution were used to assess the impact on differential cell counts. The differential cell counts for eosinophils and basophils were excluded from analysis because they were present in negligible proportions.

For each model, the possible predictors were sampling time point (pre-torpor, torpor, post-torpor), sex (male, female), body mass (g), torpor duration group (2 h, 8 h) and experiment season (autumn, winter). Sampling time point was retained in all models as a fixed effect of primary interest, reflecting our experimental design. Sex was also retained in all models as a biological covariate of interest. The others were included if they improved model fit. We used Akaike's information criterion (AIC) to assess model fit based on the parameters listed above. The model with the lowest AIC was chosen as the best fit, and when multiple models were within two AIC units, the simplest was selected. Individual ID was included as a random effect on the intercept to account for repeated measures from the same animal. Model residuals were visually inspected using *Q–Q* plots and residual versus fitted plots to further confirm that model assumptions (normality, homoscedasticity, independence and linearity) were not violated for the LMEs. GLME model assumptions (distribution and independence) were not violated. Where appropriate, Tukey's *post hoc* analyses were used to conduct pairwise comparisons using the ‘emmeans’ package (https://CRAN.R-project.org/package=emmeans). Effect sizes were quantified using Cohen's *d* within the ‘effsize’ package (doi:10.5281/zenodo.196082). Results are presented as medians±interquartile range (IQR) for total and differential WBC count, and as means±s.d. for bacterial inhibition. Model results and predictor variables included in each model are presented in Tables S1–S4.

## RESULTS

### WBC count

#### Total WBC count

**Fig. 1.**
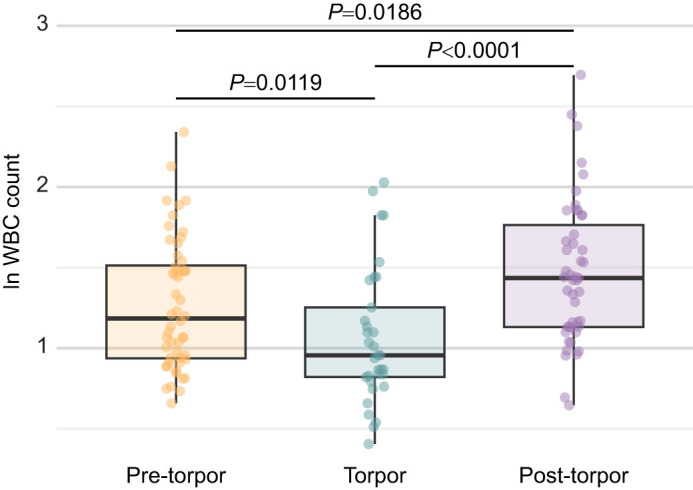
**Effect of torpor on natural log-transformed white blood cell (WBC) count in eastern bent-winged bats (*Miniopterus orianae oceanensis*) in a controlled laboratory trial.** Blood was collected from bats (*n*=52) at three time points: pre-torpor (*n*=52), during torpor (*n*=33) and post-torpor (30 min after arousal; *n*=47), and WBC count (×10^9^ l^−1^) was assessed. Boxplots display the median (thick horizontal line), interquartile range (IQR; 25th–75th percentiles; box) and values within 1.5× the IQR (whiskers).

Total WBC count differed significantly across sample collection time points (LME: χ^2^=27.79, d.f.=2, *P*<0.0001; Fig. 1). In torpor, WBC count [median±IQR: (1.60±1.22)×10^9^ cells l^−1^] was approximately 30% lower than pre-torpor values [(2.27±1.99)×10^9^ cells l^−1^; Cohen's *d*=0.54; Tukey's *post hoc* test, *P*=0.0119]. Following arousal, WBC count increased markedly [(3.20±2.75)×10^9^ cells l^−1^, Tukey's *post hoc* test, *P*<0.0001; Cohen's *d*=0.48] and exceeded pre-torpor count by 41% (Tukey's *post hoc* test, *P*=0.0186; Cohen's *d*=0.98). There were no statistically significant effects of mass, sex or season on WBC count (Table S1).

#### Differential WBC count

**Fig. 2.**
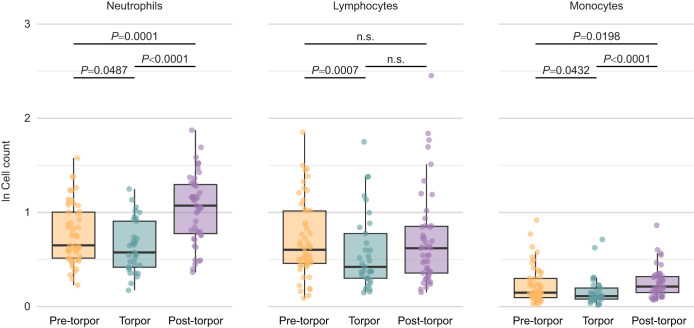
**Absolute natural log-transformed count of WBC types in the blood of Australian eastern bent-winged bats (*M. orianae oceanensis*) in torpor trials.** Blood was sampled from bats before (*n*=52), during (including animals sampled at both 2 and 8 h; *n*=33) and after (30 min after arousal; *n*=47) torpor and neutrophil, lymphocyte and monocyte count (×10^9^ l^−1^) was assessed. Boxplots show the median (thick horizontal line), IQR (25th–75th percentiles; box) and values within 1.5× the IQR (whiskers).

Neutrophils were the most common WBC type at all stages of the experiment, followed by lymphocytes and monocytes (Fig. 2).

Neutrophil numbers were significantly associated with sampling time point (GLME: χ^2^=27.48, d.f.=2, *P*<0.0001): while numbers only differed marginally between pre-torpor [median±IQR (0.91±10.5)×10^9^ cells l^−1^] and torpor samples [(0.77±0.96)×10^9^ cells l^−1^, Tukey's *post hoc* test: *P*=0.0487, Cohen's *d*=0.41], post-torpor neutrophil values were significantly higher [(1.92±1.48)×10^9^ cells l^−1^] than pre-torpor and torpor values (Tukey's *post hoc* test: *P*=0.0001, Cohen's *d*=0.81 and Tukey's *post hoc* test: *P*<0.0001, Cohen's *d*=1.15); about 111% and 150%, respectively.

Another significant predictor of neutrophil numbers was experiment season (GLME: χ^2^=3.86, d.f.=1, *P*=0.0493). Neutrophil values were about 25% higher in winter [(1.49±1.58)×10^9^ cells l^−1^] than in autumn [(1.05±1.21)×10^9^ cells l^−1^; Cohen's *d*=0.54]. Sex was not associated with changes in neutrophil count (Table S2). While band-neutrophils were screened for, they were only present in negligible amounts.

As for neutrophils, sampling time point was also a significant predictor of lymphocyte numbers (GLME: χ^2^=14.27, d.f.=2, *P*=0.0006). Values during torpor [(0.52±0.82)×10^9^ cells l^−1^] were about 40% lower than those in samples taken before torpor [(0.82±1.17)×10^9^ cells l^−1^, Tukey's *post hoc* test: *P*=0.0007, Cohen's *d*=0.25]. Post-torpor samples [(0.86±0.91)×10^9^ cells l^−1^] did not differ significantly from either pre-torpor or torpor samples. Additionally, body mass was strongly associated with lymphocyte numbers (GLME: χ^2^=11.06, d.f.=1, *P*=0.0008). For every 1 g increase in body mass, the absolute lymphocyte count increased by 0.3227 on the log scale (standard error=0.097). Sex and season were not associated with lymphocyte numbers (Table S2).

**Fig. 3.**
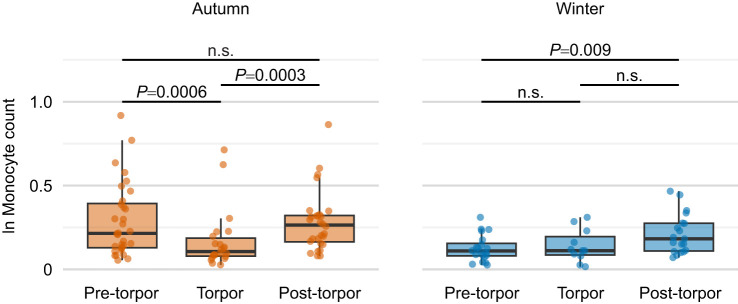
**Absolute natural log-transformed monocyte count of blood from Australian eastern bent-winged bats (*M. orianae oceanensis*) in torpor trials, according to season.** Blood was sampled from bats (*n*=52) during southern hemisphere autumn (May) and winter (July) before, during (including animals sampled at both 2 and 8 h) and after (30 min after arousal) torpor and monocyte count (×10^9^ l^−1^) was assessed. Boxplots show the median (thick horizontal line), IQR (25th–75th percentiles; box) and values within 1.5× the IQR (whiskers).

Monocyte numbers were significantly affected by sampling time point (GLME: χ^2^=21.28, d.f.=2, *P*<0.0001), and the interaction between sample and season (GLME: χ^2^=6.81, d.f.=2, *P*=0.0331; Fig. 3; Table S2). In autumn, monocyte count was significantly lower during torpor [(0.11±0.12)×10^9^ cells l^−1^] than before torpor [(0.24±0.34)×10^9^ cells l^−1^, Tukey's *post hoc* test: *P*=0.0006, Cohen's *d*=0.53] and after arousal [(0.31±0.19)×10^9^ cells l^−1^, Tukey's *post hoc* test: *P*=0.0003, Cohen's *d*=0.56], with no difference between pre- and post-arousal. In winter, monocyte count was significantly lower post-arousal [(0.20±0.20)×10^9^ cells l^−1^] than before torpor [(0.12±0.08)×10^9^ cells l^−1^, Tukey's *post hoc* test: *P*=0.0090, Cohen's *d*=0.85], while the comparison of torpor values [(0.11±0.12)×10^9^ cells l^−1^] with both pre- and post-torpor values was non-significant.

Body mass also significantly influenced monocyte count (GLME: χ^2^=4.47, d.f.=1, *P*=0.0344). For every 1 g increase in body mass, the absolute monocyte count increased by 0.167 on the log scale (standard error=0.078). Season alone and sex were not found to be associated with changes in monocyte count (Table S2).

### NLR

**Fig. 4.**
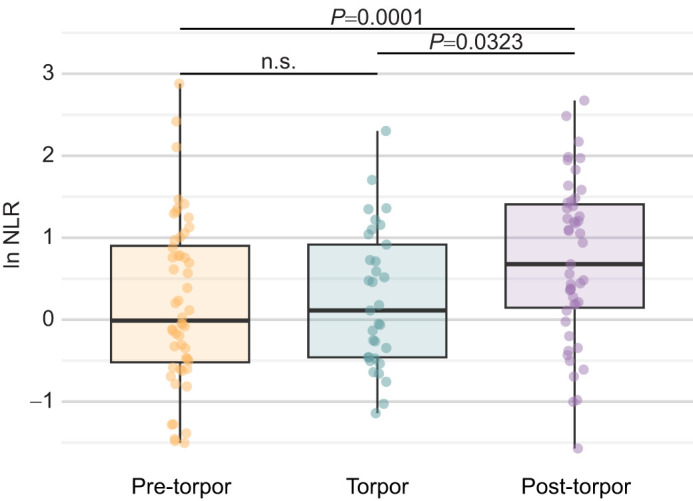
**Absolute natural log-transformed neutrophil-to-lymphocyte ratio (NLR) of blood from Australian eastern bent-winged bats (*M. orianae oceanensis*) in torpor trials.** Blood was sampled from bats (*n*=52) before, during (including animals sampled at both 2 and 8 h) and after (30 min after arousal) torpor. Boxplots show the median (thick horizontal line), IQR (25th–75th percentiles; box) and values within 1.5× the IQR (whiskers).

Sampling time point was a significant predictor of NLR (LME: χ^2^=21.00, d.f.=2, *P*<0.0001; Fig. 4; Table S3). While NLR did not differ between pre-torpor and torpor samples, mean post-torpor NLR (3.13±3.02) was about 48% higher than the pre-torpor ratio (2.11±2.98, Tukey's *post hoc* test: *P*=0.0001, Cohen's *d*=0.52), and almost 70% higher than the torpor NLR (1.86±1.93, Tukey's *post hoc* test: *P*=0.0323, Cohen's *d*=0.50). Additionally, animal body mass had a significant impact on NLR, where for each additional gram of mass, the ratio decreased by about 28% (LME: χ^2^=9.9887, d.f.=1, *P*=0.0015).

### Bacterial killing assay

**Fig. 5.**
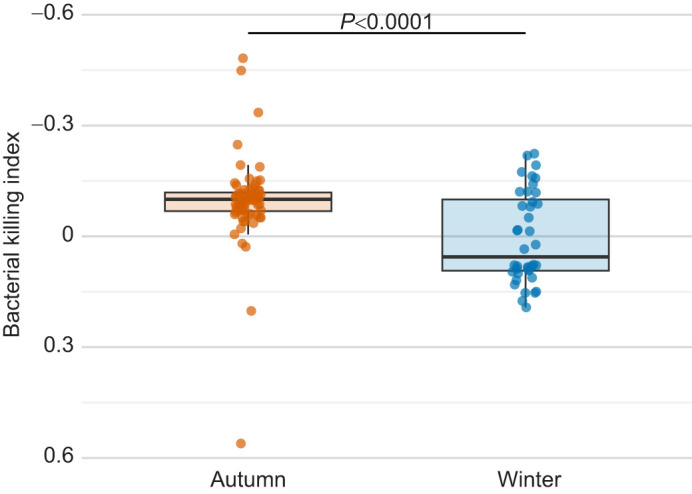
**Bacterial killing index of plasma from Australian eastern bent-winged bats (*M. orianae oceanensis*) according to season.** Blood was sampled from bats (*n*=52) during southern hemisphere autumn (May) and winter (July). Negative values indicate greater inhibition of bacterial growth. Boxplots show the median (thick horizontal line), IQR (25th–75th percentiles; box) and values within 1.5× the IQR (whiskers).

While the plasma's ability to inhibit bacterial growth was not affected by sample collection time point (pre-torpor, torpor and post-torpor; Table S4), there was a significant difference in assay values between autumn and winter (LME: χ^2^=13.71, d.f.=1, *P*<0.0001; Fig. 5). Mean bacterial inhibition capacity of plasma was about 9.5% lower in winter (0.005±0.12 index relative units) than in autumn (−0.09±1.12 index relative units; Cohen's *d*=0.82). Animal body mass was another significant predictor of bacterial inhibition assay values (LME: χ^2^=4.26, d.f.=1, *P*=0.038): for each additional gram of body mass, the bacterial killing assay value increased by about 0.03 units (standard error=0.0143), indicating a slightly weaker bacterial killing ability in the blood of heavier bats.

## DISCUSSION

Torpor profoundly alters immune function in hibernating mammals, yet most studies focus on species experiencing severe winters. Here, we investigated the impact of torpor in eastern bent-winged bats (*M. orianae oceanensis*), a species that hibernates in milder climates, providing insights into immune parameter dynamics in animals experiencing less extreme winter conditions.

The observed sharp increase in WBC count 30 min after arousal, which also surpassed pre-torpor levels, could reflect a transient overshoot in metabolic rate and body temperature during rewarming. A similar pattern has been described in ground squirrels (*Spermophilus citellus*), where circulating WBCs showed an initial spike, followed by a decline and plateau within 8 h of arousal (Bouma et al., 2010b). Although the post-arousal time frame we were able to assess was limited by the volume of blood we were able to safely collect per individual, it is likely that WBC levels in our study animals would also have returned to baseline within a few hours of arousal. Notably, our results highlight that eastern bent-winged bats can mobilise immune cells within just 30 min of arousal, whereas many other animals that employ torpor might require several hours to do so (Bouma et al., 2010a). WBC count in both hibernating and non-hibernating mammals is closely linked to body temperature (Bouma et al., 2011, 2013a,b; Sahdo et al., 2013), suggesting that the immune cell recruitment observed in our study might be directly associated with rewarming. This is further supported by the fact that the rapid recall of WBCs seen in our study follows the rapid rewarming patterns observed in other Australian insectivorous bats of similar body size (Currie et al., 2015). Studies in mice show that only about 2% of all neutrophils within the body are generally circulating in the blood stream, with additional mature cells being mobilised rapidly from the bone marrow when required (Eash et al., 2009). If the same holds true for bent-winged bats, this could explain the increase in absolute numbers of neutrophils, while no immature cells were observed.

We also found that individual types of WBCs can decrease significantly during torpor, with an especially marked reduction in the number of lymphocytes and monocytes. This finding diverges from earlier studies in other hibernators. For example, studies on Columbian ground squirrels (*Spermophilus columbianus*) (Nansel and Knoche, 1972) and thirteen-lined ground squirrels (*I. tridecemlineatus*) (Stuckey and Coco, 1942) reported an increase in lymphocytes during torpor. A likely explanation for this difference is that the previous studies investigated animals during more prolonged torpor bouts (1–6 weeks) than those examined in our study. Pilot trials (A.L., unpublished data) conducted in early 2024 showed that bats in our experimental setup remained torpid for a maximum of 8 h. Our study was therefore limited by the fact that we were unable to observe the effects of multi-day torpor. Contrary to our initial hypotheses, torpor duration also did not have a significant impact on either total or differential WBC count in our work. It is likely that the difference between the 2 and 8 h torpor bout was not long enough to observe pronounced changes in immune parameters, and that longer or repeated bouts would have been required to see an effect. This is supported by the fact that at the site bats originated from in our study, animals have recently been found to be torpid for a mean duration of about 30 h per bout (Villada-Cadavid et al., 2025). Future studies should trial further methods to optimise laboratory studies assessing long-term torpor in bent-winged bats, including a lower incubation temperature, which is generally associated with deeper torpor bouts (Geiser, 2004), and smaller respirometry chambers, mimicking the small crevices that cave-dwelling bats are often found in during winter hibernation (van Harten, 2023). We found a significant increase in the number of circulating neutrophils and monocytes upon arousal from torpor. Although we did not assess immature monocytes, the absence of increased immature (band) neutrophils suggests that elevated neutrophil numbers upon arousal are unlikely to be due to increased production. Studies on the exact mechanism of cell sequestration during torpor indicate that there are differences between cell types: while lymphocytes are stored in secondary lymphoid organs (Bouma et al., 2011), neutrophils are marginated to endothelial cells (Bouma et al., 2013a). Although monocytes have not been specifically studied in the context of sequestration in torpid mammals, it is plausible that these innate immune cells are similarly sequestered in close association with the endothelium. Assuming these principles also apply to bent-winged bats, the rapid neutrophil and monocyte response during arousal likely results from the fact that these cells essentially remain within the blood vessels, allowing a rapid non-specific response shortly after torpor. A similar shift towards a higher neutrophil percentage upon arousal has also been shown in garden dormice (*Eliomys quercinus*), where neutrophil count increased 3.6-fold compared with torpor values, while lymphocytes showed a comparatively modest rise (Huber et al., 2021). Notably, arousal in dormice was also associated with an enhanced neutrophil capacity to generate reactive oxygen species, which are essential parts of the humoral immune system that kill bacteria and fungi (Ward et al., 1988) but may also be implicated in pathological inflammation and tissue damage (Chelombitko, 2019). The NLRs that we obtained also support the strong effects of torpor state and body condition on immunity and inflammatory potential. The ratio of these cells to one another can be associated with increased glucocorticoid levels, which are indicative of physiological stress (Davis et al., 2008). If animals are in a heightened inflammatory state, neutrophils will outnumber lymphocytes and lead to a greater NLR. Consistent with our findings on arousal stimulating rapid immune recovery, NLR values were significantly higher post-torpor than during or even pre-torpor. In little ground squirrels (*Spermophilus pygmaeus*), NLR does not change significantly between baseline and post-arousal samples, which has been theorised to be aimed at minimising oxidative stress within the body (Dzhafarova et al., 2024). Eastern bent-wing bats, in contrast, appear to follow an arousal pattern similar to that of dormice, and may also produce increased amounts of reactive oxygen metabolites. Rapid immune cell recall may be beneficial, as seen in European *Myotis myotis*, in which eosinophil recruitment occurs as one component of the immune response to infection with *Pseudogymnoascus destructans*, the pathogen causing white-nose syndrome (WNS), and infection is generally survived (Bandouchova et al., 2018). However, fast recovery of immune cells upon arousal from torpor could also exacerbate pathology if the response is misdirected. In North American bats susceptible to WNS, it is hypothesised that an ineffective antifungal response and subsequent immunopathology contributes to increased arousal and subsequent death (Meteyer et al., 2012; Lilley et al., 2016; Whiting-Fawcett et al., 2021, 2026). While the pathway activated in susceptible species can support antifungal defence through neutrophil recruitment, its overactivation, as seen in heavily impacted species (Whiting-Fawcett et al., 2021), has been linked to excessive inflammation (Romani, 2001). In eastern bent-winged bats, neutrophils are rapidly recruited upon arousal, creating a heightened inflammatory potential, similar to what was observed in hibernating garden dormice (Huber et al., 2021). If an excessive misdirected antifungal response were triggered, these neutrophils could shift from protective to harmful, as an overproduction of reactive oxygen metabolites may cause host tissue damage rather than effective fungal clearance (Chelombitko, 2019). Further studies applying fungal stimuli during torpor are needed to assess how Australian hibernating bats respond to these while torpid.

Differences in leukocyte composition may also be influenced by the site of venipuncture: initial and post-torpor samples were collected from the peripheral, saphenous vein, while blood from torpid bats was collected from the central, jugular vein. If neutrophils were sequestered to the endothelium in bent-wing bats, higher post-torpor values might reflect greater retention of cells in the low-flow epithelium of a peripheral vein. However, previous work in similarly sized species has shown that there are no differences in haematological parameters between commonly used venipuncture sites in bats (Roistacher et al., 2025), suggesting minimal differences in differential cell composition across the body. Moreover, our pre- and post-torpor samples were both collected from the saphenous vein, confirming that observed increases in neutrophils and monocytes are attributable to torpor-related physiological changes rather than sampling site.

The time of year during which the experiment was conducted also had a significant impact on differential WBC count, with neutrophil percentages being overall higher in all samples collected in winter. This finding points to a seasonal variation in WBC composition. High numbers of neutrophils in winter are also observed in other hibernators such as Mongolian hamsters (*Allocricetulus curtatus*) (Kuznetsova et al., 2016) and may reflect a seasonal trade-off between specialised immunocompetence – i.e. favouring of neutrophils instead of lymphocytes – and availability of resources (Demina and Tsvey, 2022). Season further appeared to particularly influence the recovery of monocytes upon arousal. This is likely because monocyte counts were generally lower in winter, making the effects of torpor on their dynamics less pronounced than in autumn. Because monocytes represented only a small fraction of total WBCs, we saw no overall seasonal effects on total WBC count. However, the pre-torpor total WBC count in our study was slightly lower than that reported in a related species, the southern bent-winged bat (*Miniopterus orianae bassanii*), which had a mean (±s.d.) count of (5±3.1)×10^9^ cells l^−1^ (Holz et al., 2020). This discrepancy likely reflects differences in sampling season: the earlier study sampled bats in September (southern hemisphere spring), a time when bats are emerging from hibernation and WBC count is expected to rise as energy availability increases (Graesli et al., 2015; Bouma et al., 2010a,b). It is possible that repeating our experiment in spring would have revealed stronger seasonal effects, particularly in terms of immune cell recovery, which may accelerate with increasing metabolic activity.

Torpor did not affect the results of our bacterial killing assay. This is unsurprising because immune effectors measured by this method are innate and do not rely on continued provision of metabolic energy to function. However, our bacterial killing assay assessed humoral components of innate immunity and does not capture cellular immune processes such as phagocytosis. Phagocytosis is more energetically demanding and is typically suppressed during torpor, but, in line with rapid WBC recall, has been shown to be rapidly re-established following arousal in hibernating bats (Nemcova et al., 2022). Thus, the absence of a torpor effect in our bacterial killing assay should not be interpreted as evidence that all innate immune functions are maintained during torpor. Gram-negative bacteria such as the strain of *E. coli* used in our assay are killed by the complement system. The complement system is a part of the humoral innate immune system comprising various proteins that can both facilitate and directly induce pathogen lysis (Sarma and Ward, 2011). The function of the complement system can decrease either quantitatively, as a result of reduced concentrations of active molecules during torpor, or qualitatively, as a result of the lower efficiency of biochemical reactions at colder temperatures (Bouma et al., 2010a). This is important to differentiate as our assay was conducted at 37°C, which reflects the active body temperature of bats rather than the reduced body temperature typical of torpor. Measuring bacterial inhibition/killing ability at 37°C therefore assesses the functional potential of existing humoral components under optimal physiological conditions, rather than their activity at torpid body temperatures. Previous work on golden-mantled ground squirrels (*Callospermophillus lateralis*) found that serum complement levels decrease in torpor (Maniero, 2002). However, the study also noted a decrease proportional to torpor length, with samples taken after short torpor bouts (24–36 h) showing higher levels of complement activity than those taken after long bouts (longer than 7 days). While short torpor bouts like those in our experiment appear not to substantially reduce humoral antimicrobial function in eastern bent-winged bats at active body temperature, longer bouts may cause measurable declines because of the degeneration of complement factors over time (quantitative reduction rather than reduction of biochemical potential). This is consistent with our findings on seasonal differences in bacterial inhibition/killing activity. Furthermore, complement activity in golden-mantled ground squirrels was similarly higher in summer to that in any of the winter samples. It has been shown that the production of other components of the humoral immune system, namely signal proteins known as cytokines, is negatively affected by fasting (Novoselova et al., 2000). Low energy availability is also likely to reduce the production of complement proteins, which would further reduce the levels of these factors. This is consistent with our observation that mid-winter bacterial killing ability was lower in our animals as well, as less food would have been available to them. Interestingly, we observed a negative relationship between initial body mass measured and results of our bacterial killing assay, with heavier bats showing lower bacterial killing ability values. This negative relationship could reflect energetic trade-offs, whereby bats in better body condition allocate resources to other physiological processes, such as cellular rather than humoral immunity. Metabolic costs of the complement system are low compared with induced, cell-mediated responses (Lee, 2006). Bats in poorer body condition may therefore allocate proportionally more energy toward innate immune processes, whereas bats with higher body mass exhibit comparatively higher WBC counts and, as indicated by lower NLR values, may have greater energetic capacity to support adaptive immune responses.

To summarise, our study provides new insights into immune parameters of a southern hemisphere bat species that uses winter hibernation, highlighting changes that occur during short-term torpor and arousal. We found that torpor induces a significant but reversible reduction in total WBC count, consistent with patterns observed in hibernating mammals in the northern hemisphere. Upon arousal, neutrophils and monocytes are mobilised first, which might offer immediate protection against pathogens encountered but also carries the potential for immunopathology if excessively activated. In contrast, humoral innate immune components, specifically the complement system, remained stable during short torpor bouts. Our results also highlight the strong influence of season on both plasma and cellular immune components, likely affecting the bats' ability to respond to pathogens throughout the year. These findings provide a critical baseline for evaluating the susceptibility of southern hemisphere bats to emerging pathogens, including the potentially fatal white-nose syndrome. Future research should investigate how prolonged torpor, seasonal shifts and pathogen-specific challenges interact to influence immune capacity and predicted disease outcomes in these species.

## Supplementary Material

10.1242/jexbio.251962_sup1 Supplementary information
