# Supplementary material for: Emergence from torpor rapidly elevates suppressed blood immune parameters in a bat species hibernating in a moderate climate
Source: J Exp Biol. 2026 May 11;229(9):jeb251962. doi: 10.1242/jeb.251962 (PMC13245917; doi:10.1242/jeb.251962)
Supplement: Supplementary information [file jexbio-229-251962-s1.pdf]

Table S1. Absolute white blood cell counts. Results of linear mixed-effects models (LMEs) assessing predictors of total white blood cell counts in 52 eastern bent-winged bats (*Miniopterus orianae oceanensis*) enrolled in a controlled laboratory trial to assess the effects of torpor status on immune parametres. Fixed effects in the model of best fit included sampling timepoint (pre-torpor, in torpor, post-torpor), body mass (g), sex (male, female), and experiment season (autumn, winter). Random intercepts for individual Bat ID account for repeated measures. Test statistics (χ^2), degrees of freedom (df), and p-values are presented for each fixed effect. Significant *p* values are indicated with asterisks: p < 0.001 (***).

Sample size	Statistical model used	Fixed effects	Random effects	Random effects model result	Test statistic	Df	P value
N = 132 (52 individuals)	LMER	Sample			χ^2 = 27.4803	2	< 0.001 ***
		Body mass			χ^2 = 3.2144	1	0.0730
		Sex			χ^2 = 0.3019	1	0.5827
		Season			χ^2 = 0.151	1	0.9022
			Bat ID	Variance = 0.11818 ± 0.3438			

Journal of Experimental Biology • Supplementary information

Table S2. Differential white blood cell counts. Results of generalised linear mixed-effects models (GLMs) assessing predictors of differential white blood cell counts in 52 eastern bent-winged bats (*Miniopterus orianae oceanensis*) enrolled in a controlled laboratory trial to assess the effects of torpor status on immune parametres. Fixed effects included in the respective models of best fit are shown below and included sampling timepoint (pre-torpor, in torpor, post-torpor), body mass (g), sex (male, female), and experiment season (autumn, winter), as well as their interactions. Random intercepts for individual Bat ID account for repeated measures. Test statistics (χ^2), degrees of freedom (df), and p-values are presented for each fixed effect. Significant *p* values are indicated with asterisks: p < 0.05 (*), p < 0.001 (***).

Output variable	Sample size	Statistical model used	Fixed effects	Random effects	Random effects model result	Test statistic	Df	P value
Absolute neutrophile counts	N = 132 (52 individuals)	GLM	Sample			$\chi^2 = 39.1532$	2	< 0.001 ***
			Sex			$\chi^2 = 0.1261$	1	0.7225
			Season			$\chi^2 = 3.8648$	1	0.0493 *
				Bat ID	Variance = 0.05644 ± 0.2376			
Absolute lymphocyte counts	N = 132 (52 individuals)	GLM	Sample			$\chi^2 = 14.7244$	2	< 0.001 ***
			Body mass			$\chi^2 = 11.0662$	1	< 0.001 ***
			Sex			$\chi^2 = 1.3425$	1	0.2466
			Season			$\chi^2 = 0.4268$	1	0.5135

Journal of Experimental Biology • Supplementary information

				Bat ID	Variance = 0.3615 ± 0.6013			
Absolute monocyte counts	N = 132 (52 individuals)	GLM	Sample			$\chi^2 = 21.2845$	2	< 0.001 ***
			Body mass			$\chi^2 = 4.4739$	1	0.0344 *
			Sex			$\chi^2 = 1.8067$	1	0.1789
			Season			$\chi^2 = 1.7104$	1	0.1909
			Sample : Season			$\chi^2 = 6.8156$	2	0.0331 *
				Bat ID	Variance = 0.1949 ± 0.4415			

Journal of Experimental Biology • Supplementary information

Table S3. Neutrophil-to-Lymphocyte ratio. Results of linear mixed-effects models (LMEs) assessing predictors of neutrophil-to-lymphocyte ratio in 52 eastern bent-winged bats (*Miniopterus orianae oceanensis*) enrolled in a controlled laboratory trial to assess the effects of torpor status on immune parametres. Fixed effects included in the model of best fit were sampling timepoint (pre-torpor, in torpor, post-torpor), body mass (g), sex (male, female), and experiment season (autumn, winter). Random intercepts for individual Bat ID account for repeated measures. Test statistics (χ^2), degrees of freedom (df), and p-values are presented for each fixed effect. Significant *p* values are indicated with asterisks: p < 0.01 (**), p < 0.001 (***).

Sample size	Statistical model used	Fixed effects	Random effects	Random effects model result	Test statistic	Df	P value
N = 132 (52 individuals)	LMER	Sample			$\chi^2 = 21.0055$	2	< 0.0001 ***
		Body mass			$\chi^2 = 9.9877$	1	0.001575 **
		Sex			$\chi^2 = 1.3834$	1	0.2395
		Season			$\chi^2 = 12.8615$	1	0.0907
			Bat ID	Variance = 0.4220 ± 0.6496			

Table S4. Bacterial inhibition index. Results of linear mixed-effects models (LMEs) assessing predictors of bacterial inhibition index in 52 eastern bent-winged bats (*Miniopterus orianae oceanensis*) enrolled in a controlled laboratory trial to assess the effects of torpor status on immune parametres. Fixed effects included in the model of best fit were sampling timepoint (pre-torpor, in torpor, post-torpor), body mass (g), sex (male, female), and experiment season (autumn, winter). Random intercepts for individual Bat ID account for repeated measures. Test statistics (χ^2), degrees of freedom (df), and p-values are presented for each fixed effect. Significant *p* values are indicated with asterisks: $p < 0.01$ (**), $p < 0.001$ (***).

Sample size	Statistical model used	Fixed effects	Random effects	Random effects model result	Test statistic	Df	P value
N = 132 (52 individuals)	LMER	Sample			$\chi^2 = 5.2452$	2	0.0726 *
		Body mass			$\chi^2 = 4.2677$	1	0.0388 *
		Sex			$\chi^2 = 1.4689$	1	0.2255
		Season			$\chi^2 = 13.9851$	1	0.0002 ***
			Bat ID	Variance = 0.006387 ± 0.07992			

Journal of Experimental Biology • Supplementary information
